# Analysis of spatial patterns and driving factors of provincial tourism demand in China

**DOI:** 10.1038/s41598-022-04895-8

**Published:** 2022-02-10

**Authors:** Xuankai Ma, Zhaoping Yang, Jianghua Zheng

**Affiliations:** 1 College of Resource and Environment Sciences, Xinjiang University, Ürümqi, 830046 China; 2 Xinjiang Institute of Ecology and Geography, Chinese Academy of Sciences, No.818, Beijing South Road, Ürümqi, 830011 Xinjiang China; 3 University of Chinese Academy of Sciences, Beijing, 100049 China

**Keywords:** Environmental economics, Socioeconomic scenarios, Sustainability

## Abstract

Modeling and forecasting tourism demand across destinations has become a priority in tourism research. Most tourism demand studies rely on annual statistics with small sample sizes and lack research on spatial heterogeneity and drivers of tourism demand. This study proposes a new framework for measuring inter-provincial tourism demand's spatiotemporal distribution using search engine indices based on a geographic perspective. A combination of spatial autocorrelation and Geodetector is utilized to recognize the spatiotemporal distribution patterns of tourism demand in 2011 and 2018 in 31 provinces of mainland China and detect its driving mechanisms. The results reveal that the spatial distribution of tourism demand manifests a vital stratification phenomenon with significant spatial aggregation in the southwest and northeast of China. Traffic conditions, social-economic development level, and physical conditions compose a constant and robust interaction network, which dominates the spatial distribution of tourism demand in different development stages through different interactions.

## Introduction

Tourism is an essential driver of world economic development. The world was affected by the COVID-19 outbreak in 2019, and according to a report by the World Tourism Organization^1^, the world's top ten consumer countries showed continued growth in tourism consumption against the backdrop of a global economic slowdown. According to the Ministry of Culture and Tourism of the People’s Republic of China (https://www.mct.gov.cn/), China's 2020 annual domestic tourism numbered 2.879 billion trips, down 52.1% from a year earlier. The first quarter of 2021 saw 11.872700 billion domestic trips organized by national travel agencies, increasing 138.50% year-on-year. As the contribution of tourism to the regional economy is improving more and more significantly, the study of tourism demand has become a popular research topic^2^. Many scholars have carried out tourism demand forecasting through qualitative analysis, time series models, econometric models with artificial intelligence, and the accuracy of forecasting has gradually improved. However, tourism and tourists are closely correlated in terms of spatial mobility, and if spatial effects are ignored, a model estimation can be biased and produce misleading coefficient estimates^3–5^. Deng and Athanasopoulos were the first to incorporate spatiotemporal dynamics into an Australian domestic tourism demand model study^6^, and Yang and Zhang showed that spatiotemporal models have a significantly enhanced effect on the performance of tourism demand forecasting between domestic provinces in China^7^. Liu et al. noted that demographic factors, climate, key transportation modes, economic level, and other aspects of tourism demand were not investigated^8^. Therefore, it is imperative to understand the spatiotemporal patterns and driving mechanisms of tourism demand.

From the tourism supply side, the geographical and spatial clustering of tourism-related services produces spatial dependence and scale effects at the macro level, thus providing tourists with more acceptable prices and convenient services to achieve regional tourism growth. From the tourism demand side, tourists from the same region have more similar social psychology and tourism demand^9^, their tourism demand patterns are similar in terms of spatial preferences, and travel patterns show a more consistent cyclicality in time. Domestic tourism is an undisputed driver of economic development and poverty alleviation in less developed regions than international tourism^10^. Pompili et al. argued that choosing the provincial level as the geographic unit to study tourism flows yields more valuable results^11^. The results of the detection of spatial effects within a region can provide a scientific and empirical reference to local governments, tourism planners and administrative units regarding resource allocation and infrastructure development.

Several studies have explored the factors affecting tourism demand. For example, Priego et al. explored the impact of climate change on domestic tourism flows in Spain^12^. Massidda and Etzo studied the contribution of road infrastructure to tourism demand in domestic tourism in Italy^9^. Priego et al. emphasize the importance of meteorological factors on domestic travel destinations in Spain^12^. Technological innovation^13^ and knowledge spillovers^14^ cannot be ignored in driving tourism productivity and making tourism demand grow. Alvarez‐Diaz et al.^15^, Marrocu and Paci^16^, Massidda and Etzo^9^ confirmed that the size of the population is also one of the drivers of tourism demand. Despite the large number of studies exploring the factors affecting tourism demand, most researchers have focused more on the impact of single aspects of socio-economic or natural factors on tourism demand, and there are no studies based on a geographic perspective that integrate the various dominant factors into a comprehensive mechanism of impact on tourism demand.

We found from the early literature that the number of tourists and tourism income served as the main proxies for tourism demand modeling. With the development of the Internet, some researchers^8,17^ found that tourists' search engines for tourism information retrieval are the starting point and an essential part of tourism decision and travel. Li et al. summarized relevant 2012–2019 in their latest review^18^. We know about search engine data primarily based on empirical studies investigating the eximious contribution to tourism demand observation and forecasting. With Google Trends being widely used for tourism demand forecasting at multiple spatial scales worldwide^19^, Baidu Index performs even better in the Greater China region^20^. Song et al. demonstrated that Internet data has a significant driving effect on tourism demand research, with search engine data being the most common Internet data source used by researchers^19^. It is now well established from various literature that analytical methods have been implemented to address the single driving mechanisms of tourism demand. In their paper, Marrocu and Paci indicated that the application of spatial autoregressive models gave the spatial dependence patterns of tourism flows access to be effectively presented^16^. Yang and Fik investigated tourism growth change in 342 cities in China using spatial growth regression models^21^. Deng et al. used a spatial econometric analysis framework to analyze the impact of air pollution on inbound tourism in China^22^. In general, tourism demand is not affected by any individual factor, and the interactions among the factors affect the distribution of tourism demand. Therefore, it is crucial to detect the interrelated effects of tourism demand drivers. However, most existing studies ignore the interaction among the drivers of tourism demand. In addition, most existing models used in the literature make assumptions about the data and fail to reveal the interaction among the factors.

To fill these gaps, this study aims to address the spatial heterogeneity and drivers of tourism demand by using 678,900 Origin–Destination flows (OD flows) of tourism demand data from 31 Chinese provinces at the years 2011 and 2018, which helps gain insight into the spatial heterogeneity of tourism demand exhibited in the period of rapid economic development. Second, to our knowledge, this might be the first attempt to present a theoretical framework for a multi-factor driving mechanism of tourism demand, which incorporates social-economic development, population size, urban ecological conditions, tourism resources, physical conditions, traffic conditions, and technological innovation. Third, from the perspective of spatially stratified heterogeneity, this study taps the influence of the main driving factors and the interaction between different potential factors on the spatial heterogeneity of tourism demand. In addition, the study of tourism demand should not only focus on the influence of local economic activities and the natural environment but also the influence of inter-regional spatial correlation. Therefore, this study uses spatial autocorrelation and geographic detector models to analyze the spatial variation of tourism demand and its drivers at the provincial level.

The rest of the study is organized as follows: “Materials and methodology” section provides an overview of the proxies affecting tourism demand and the data and methods used in this paper. “Results” section analyzes the drivers and spatial characteristics of tourism demand. Finally, “Discussion” and “Conclusions” sections are the discussion and conclusion of the findings, respectively.

## Materials and methodology

### Study areas

**Figure 1:**
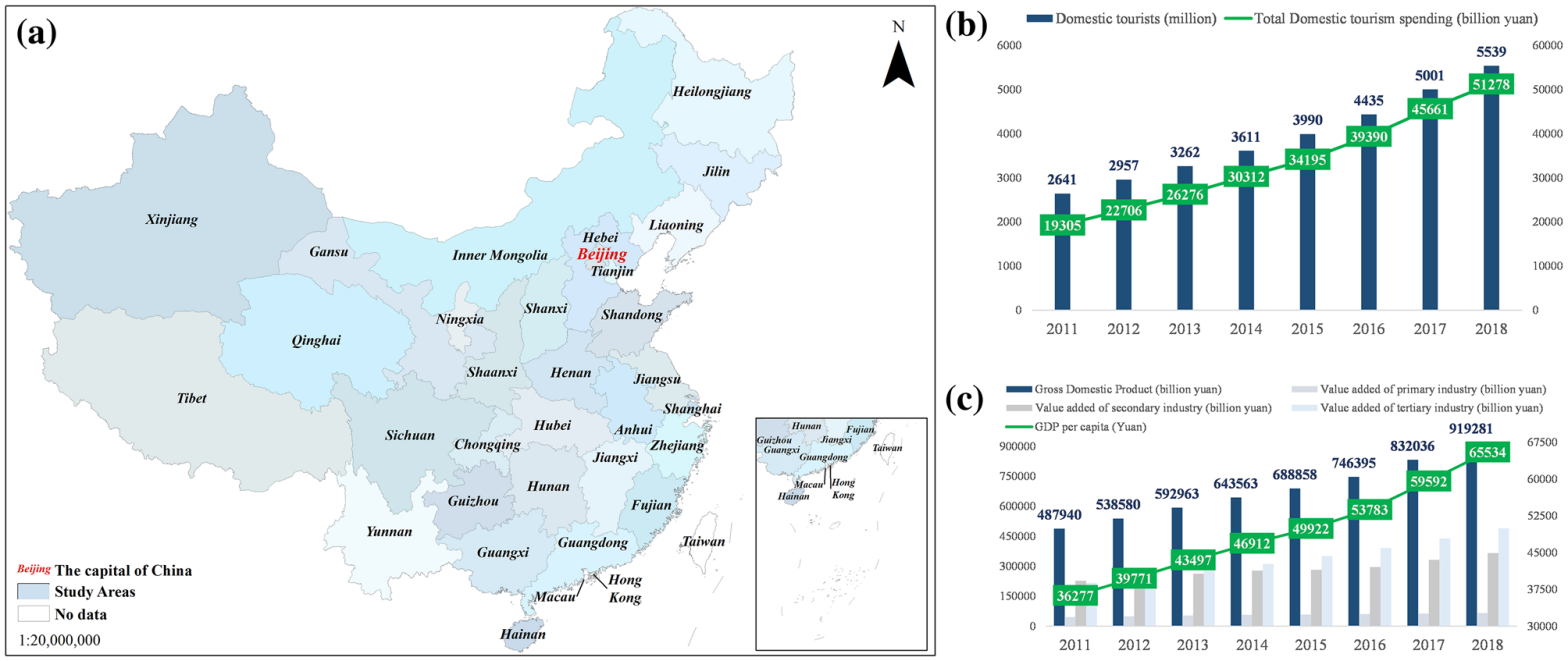
An overview map of the study area: (**a**) 31 provinces in mainland China; (**b**) economic conditions in the study area from 2011–2018; (**c**) tourism in the study area from 2011 to 2018. Data on China's economy are from the National Bureau of Statistics of China (http://www.stats.gov.cn/) and the Chinese Academy of Social Sciences (http://english.cssn.cn/). Standard map services are provided by the Ministry of Natural Resources of China (http://bzdt.ch.mnr.gov.cn/), GS (2020)4619.

After the world economic crisis in 2008, China started its economic recovery in 2011, followed by an average annual growth of 9.48% in GDP and 14.99% in total domestic tourism consumption until 2018 (Fig. 1b,c). This paper investigates the factors driving the changes in the spatial distribution of tourism demand at a provincial level in China in 2011 and 2018 to provide a basis for planning decision-makers in developing countries and regions. This study regarded the provinces as the primary geographical unit, and 31 administrative provinces in mainland China were selected as the study area. Due to the unavailability of data, Hong Kong, Macao, and Taiwan are not included in the study area (Fig. 1).

### Dominant factors and proxy variables of tourism demand

**Figure 2:**
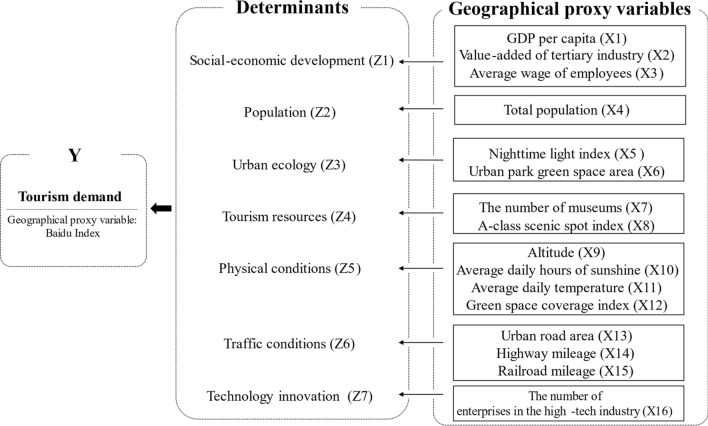
Determinants and their geographical proxy variables concerning the spatial distribution of tourism demand.

From a systemic perspective, the underlying driving mechanism of tourism demand is constituted by the tourist travel intention of the source and the destination's attractiveness. It is influenced by the resistance of temporal distance, spatial distance, and social distance^23–25^. Natural conditions and human factors determine tourism demand (Fig. 2). In this study, social-economic development (Z1), population (Z2), urban ecology (Z3), tourism resources (Z4), physical conditions (Z5), traffic conditions (Z6), and technology innovation (Z7) are used as factors that directly affect tourism demand. Considering the availability of data, GDP per capita, value-added of tertiary industry, and the average wage of employees is used to characterize the level of socio-economic development. The total population and nighttime light index measure the population scale. Urban ecological condition is indicated by Urban park green area. Museums and A-class scenic spot index represent the richness of tourism resources. The physical environmental conditions consist of altitude, average daily hours of sunshine, average daily temperature, green space coverage index. Transportation conditions are reflected by the urban road area, highway mileage, and railroad mileage. The number of enterprises in the high-tech industry represents the region's scientific and technological innovation capability. The search intensity of the Baidu index was employed to quantify tourism demand.

### Data

#### Tourism demand of inter-province

**Figure 3:**
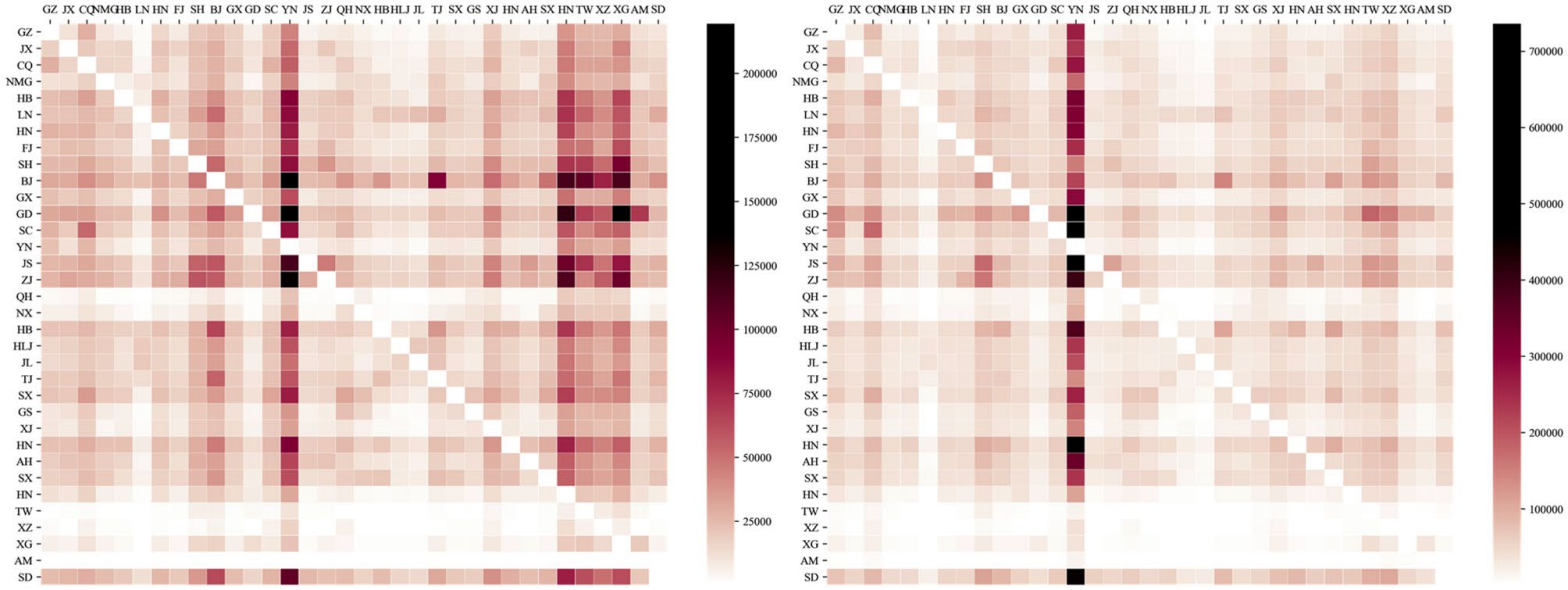
Correlation matrix of tourism demand between provinces: Based on the daily Baidu indexes accumulated throughout the year in (**a**) 2011; (**b**) 2018. The Y-axis is the origin, and the X-axis is the abbreviation that replaces the destination, the name of each province. The color of each grid represents the total annual amount of tourism demand from the source province to the destination province. The grid color From lighter to darker represents the strength of tourism demand flow.

Search engine big data are one of the data sources that can accurately quantify tourism demand. Search engines collect records of Internet users retrieving information on the Internet to form search engine indices with high timeliness. Baidu index (https://index.baidu.com/) has better accuracy in the Greater China region for measuring tourism demand, and keywords query it. The keyword database consisted of the combinations of destination provinces name + "tourism". The Baidu index of each keyword could be decomposed according to the region and time, to obtain the daily search intensity of internet users in province A for tourism information in province B. We constructed an origin–destination (OD) spatiotemporal matrix in 2011 and 2018, which contains the intensity of travel information retrieved by residents of one province on the Internet for another province for each day. The correlation matrix of tourism demand among provinces visualized in Fig. 3 was obtained by summing up the daily tourism demand flows by year and accumulated in terms of destination provinces to obtain the tourism retrieval index of each province for a year. It characterized the total tourism demand of that province. Additionally, the annual Baidu Index of province-A Internet users query for tourism information about province-B is defined as a tourism demand flow for this OD.

#### Indicators for influencing factors

We collected statistical panel data released in 2012 and 2019 from the China City Statistical Yearbook (http://www.stats.gov.cn/tjsj/tjcbw/), including official statistics on the economic development level, population, urban ecological conditions, transportation conditions, and science and technology innovation. A-class tourism resources lists were extracted from the summary of government documents published on each local government website, and the tourism resources were numerically mapped according to A-1, AA-2, AAA-3, AAAA-4, and AAAA-5 remapping. The tourism resource addresses were geocoded into spatial point data, and kriging interpolation was implemented for spatial interpolation to generate the A-class tourism resources index raster. In addition, we used remote sensing data as a geographic proxy variable for physical conditions and population distribution. The NPP-VIIRS-like NTL Data from Harvard Dataverse (https://library.harvard.edu/services-tools/harvard-dataverse/), which represents the intensity of human activity at night, the elevation data is from the Resource and Environmental Science Data Center of the Chinese Academy of Sciences (https://www.resdc.cn/), the climate data is from the National Meteorological Science Data Center of China (http://data.cma.cn/), and the green space coverage index is from the USGS (https://www.usgs.gov/). All proxy variables were free of being counted according to provincial administrative boundaries, and the raw data were in a GeoDetector model where sampling points would capture the values of different variables.

### Methodology

#### Exploratory spatial data analysis

Exploratory spatial data analysis is a series of spatial data statistical analyses applied to describe and visualize the spatial and temporal distribution patterns of tourism demand. Global spatial autocorrelation^26^ is adopted to determine whether the spatial distribution pattern of tourism demand is clustered, dispersed, or random^27^. The local spatial autocorrelation^28^ is practiced in identifying areas where spatial clustering and outliers occur to explore their spatial effects^29^. Considering the spatial data of provinces are polygons and checked by topology, Queen contiguity is utilized to indicate the spatial weight matrix between provinces^30^.

#### Spatial stratification heterogeneity analysis

GeoDetector is an advanced spatial statistical analysis model used to study factors' impact on diseases at a specific geographical area early^31^. Furthermore, it gradually developed into various research fields with spatial characteristics, such as ecological security, food production, urban land use, carbon emissions. It is a hypothesis that if the independent variable directly influences the dependent variable in space, then the spatial distribution of the dependent variable should converge with the spatial distribution of the independent variable^32^. The model detects the similarity of two variables in spatial distribution patterns from the perspective of spatially stratified heterogeneity^33^.

**Figure 4:**
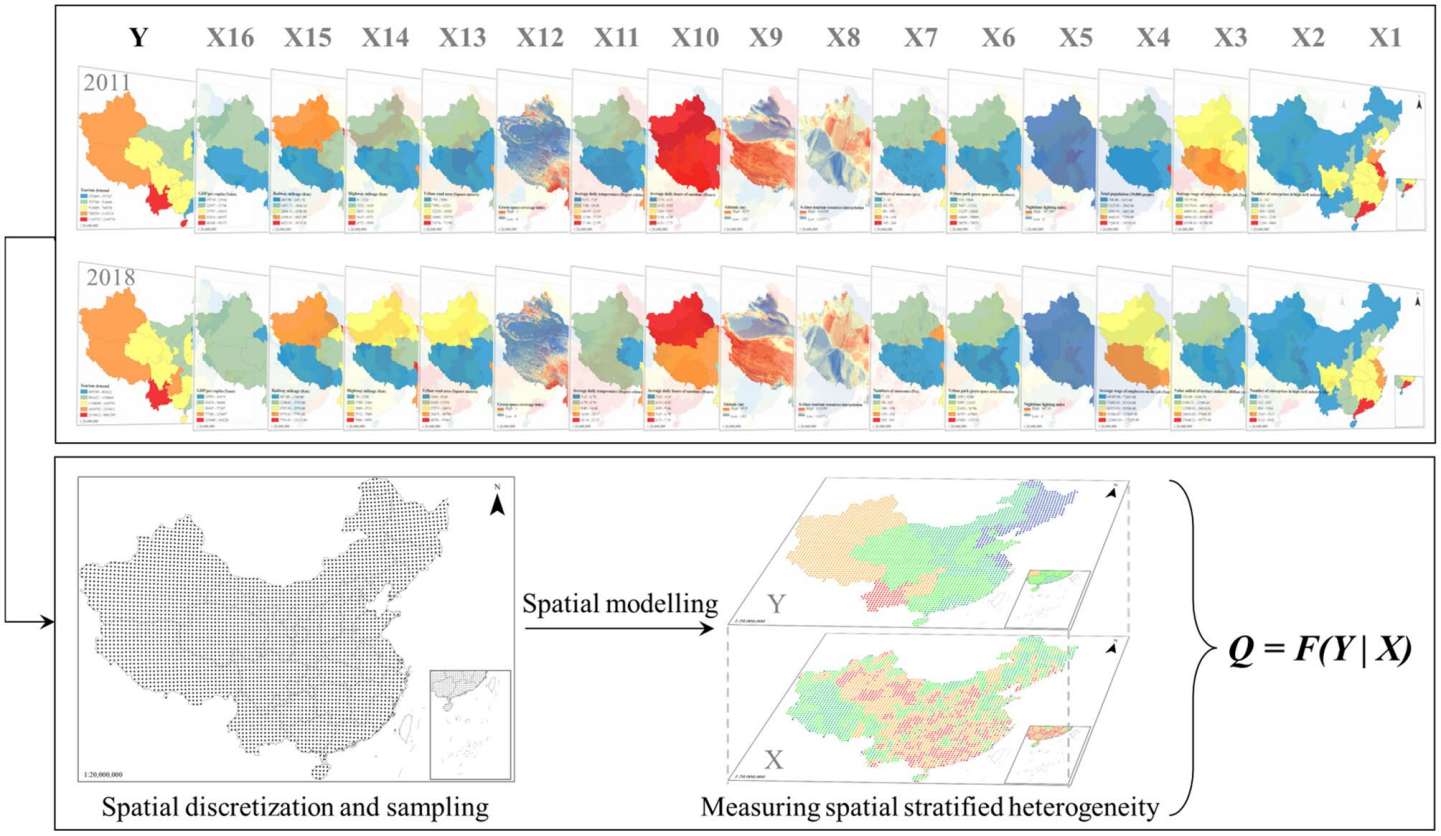
Principle of geodetector.

In this paper, GeoDetector was adopted to analyze the factors affecting the spatial distribution of tourism demand in 31 provinces of China (Fig. 4). Factor detection, ecological detection, and interactive detection submodules were applied to quantify their spatial heterogeneity and interactions between factors on the spatial distribution of tourism demand. The Q statistic measured and explained the influence of the independent variable X on the dependent variable Y on spatial heterogeneity. The expressions are as follows.

1 $$Q=1-\frac{\sum_{j=1}^{M} {N}_{j}{\sigma }_{j}^{2}}{N{\sigma }^{2}}$$

where: $Q$-statistic is a measure of the explanatory power of the influence of factor X on tourism demand Y; M represents the number of strata (subdivisions); $N$ represents the number of provincial geographical units in the study area; ${N}_{j}$ represents the number of provinces in subdivision $j$; ${\sigma }^{2}$ and ${\sigma }_{j}^{2}$ indicate the variance of tourism demand in the whole study area and the variance of tourism demand in each subdivision, respectively. The greater the value of Q, the stronger the influence of factor X on tourism demand Y.

Stratification of geographic proxy variables has a significant impact on the accuracy of factor detection. The optimization algorithm for stratifying geographic proxy variables parameters proposed by Song et al. offered optimizing spatial discretization^34^. The optimization algorithm assumes that each variable is stratified using different unsupervised discretization methods to form different stratification schemes. If one alternative scheme obtains an enormous Q-statistic based on factor detection calculations, this stratification scheme captures the most significant driving force between that variable and the observed variables.

The study area was spaced at 50 km intervals, and 3795 sampling points were generated to sample 16 continuous-type variables. Quantile method, natural break method, geometric break method, standard deviation break method, and equivalence breakpoint method were used as statistical stratification methods with intervals of 3–6, and the Q-statistic of proxy variables and observations under different stratification schemes were probed. Finally, the scheme with the enormous Q-statistic was selected as the stratification and interval parameters for this proxy variable.

## Results

### Spatial patterns of tourism demand in provinces

#### Spatial distribution

**Figure 5:**
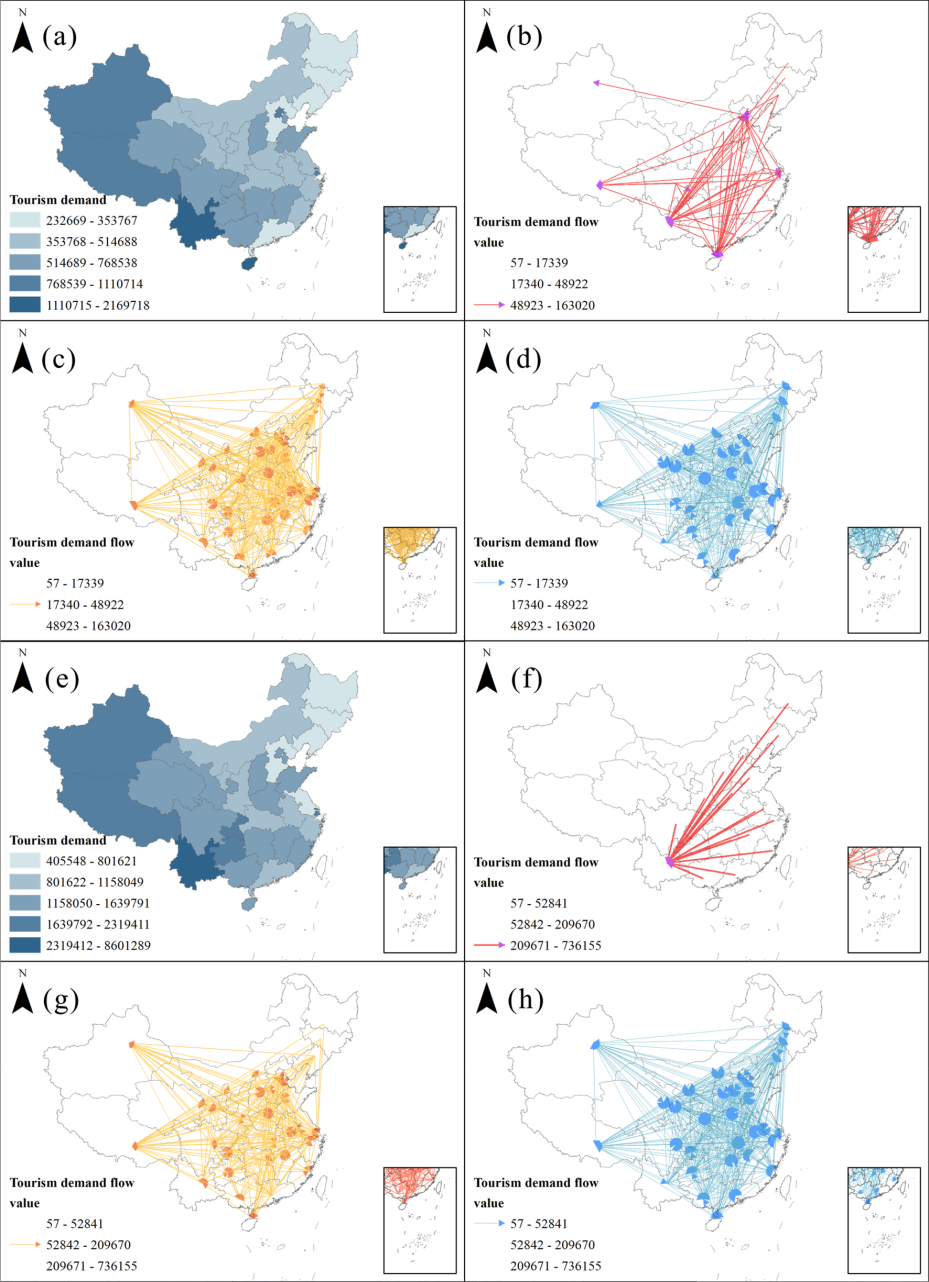
Spatial distribution of tourism demand and tourism demand flow: (**a**) tourism demand in 2011, and (**e**) is in 2018; (**b**–**d**) are the inter-provincial directional tourism demand flows in 2011, which are high-intensity flow, medium intensity flow, and low-intensity flow respectively, and (**f**–**h**) is in 2018.

Figure 5 illustrates the spatial distribution of tourism demand and flows between provinces in 2011 and 2018. In 2011, tourism demand was mainly distributed in China's first-tier cities with Beijing and Shanghai and the border provinces from southwest to northwest, with Hainan, Yunnan, Tibet, and Xinjiang being the main tourism demand destinations 35.33% of tourism demand in the study area. In 2018, tourism demand showed a trend of migration to first-tier cities, with Yunnan, Tibet, Shanghai, Chongqing, and Guizhou accounting for 36.46% of the total tourism demand.

From the spatial perspective of tourism flow, there are 930 tourism flows between provinces with a minimum Euclidean distance of 115 km, a maximum of 3600 km, and a median of 1286 km. Long-distance travel^35^ is a characteristic of tourism between domestic provinces. In 2011 the tourists' origins were concentrated in eastern China, and there were two clusters of high-intensity tourism flows distributed from Beijing and the ring-Beijing area to Yunnan and Hainan; this phenomenon altered significantly in 2018, with high-intensity tourism flows concentrated on one cluster of tourism flows from eastern China to Yunnan Province. Medium-intensity tourism flows presented a complex network with a stochastic pattern in 2011; in 2018, the complexity of the tourism network decreased, with steady clusters of tourism flows originating from the eastern provinces to Xinjiang and Tibet, while the northeastern region became a complete exporter of tourists. Comparing the low-intensity tourism flows in 2011 and 2018, it can be attended that the overall origin and destination were practically completely connected, i.e., there were tourism flows from both border provinces and the central region. The central provinces also energetically export tourists to the peripheral provinces, diverting the overall tourism flow network to a more luxuriant state. In summary, we can catch that the domestic tourism network in China during the period of rapid economic development showed a remarkable complex pattern, with the origins of tourists consolidated in the densely populated and economically developed areas in the east and the destinations distributed in the first-tier cities, and remote areas in the central and western regions.

**Figure 6:**
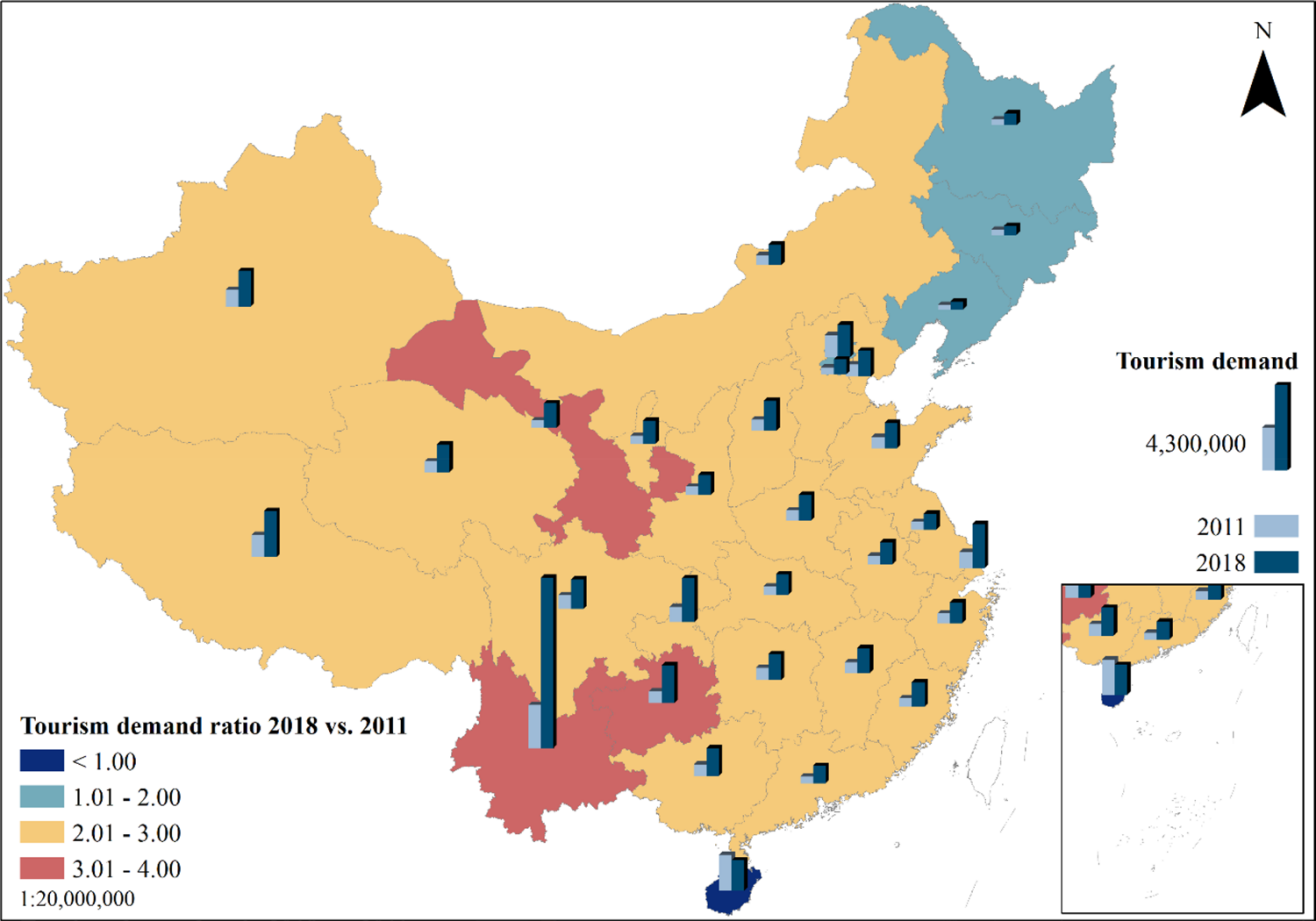
Spatial distribution of the ratio of tourism demand in 2018 to 2011.

From 2011 to 2018, the northeastern provinces, Beijing-Tianjin-Hebei region (Beijing, Tianjin, Hebei), Yangtze River Delta region (Shanghai, Jiangsu, Zhejiang, Anhui), and Pearl River Delta region (Guangzhou) are stable tourist sources; the average value of tourism demand in each province rose from 642,515 to 1,529,387, an increase of 2.38 times (Fig. 6). Yunnan and Guizhou in the southwest and Gansu in the west grew at a much higher rate than the national average; Heilongjiang, Jilin, and Liaoning in the northeast grew at a much lower rate than the average; and Hainan in the south became the only province in the country where tourism demand decreased.

#### Spatial dependency

The spatial distribution pattern of tourism demand shifted from medium to high clustering in 2011 and 2018, and the positive Moran’s I revealed the existence of high-value to high-value clustering or low-value to low-value clustering of tourism demand in the study area, and the spatial pattern and spatial dependence of tourism demand with evident clustering. The z-score increased by 177.58% during this period, and the p-value decreased by 88.96%. The probability of rejecting the null hypothesis increased from 90 to 95%. Thus, the spatial clustering trend of tourism demand strengthened.

**Figure 7:**
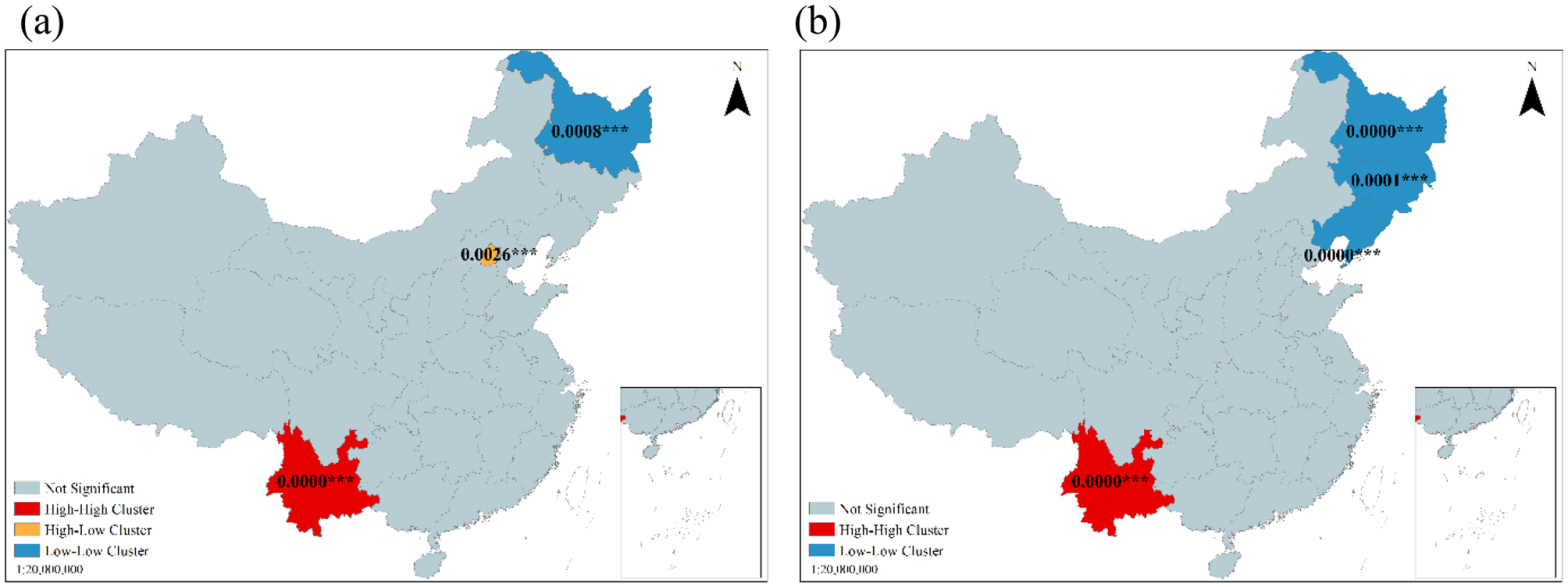
Local indicators of spatial association (LISA) for tourism demand in (**a**) 2011 and (**b**) 2018. The numerical marks on the maps represent P values, ** 5% level of significance (P < 0.05); *** 1% level of significance (P < 0.01).

The global Moran’s I identified the overall spatial dependence of tourism demand in each province within the study area, and Local spatial autocorrelation analysis was applied to uncover the local spatial association patterns. As can be seen from Fig. 7, significant stratification of tourism demand in 2011 and 2018 on a local spatial basis in China (absolute value of Z-score > 2.56, p-value < 0.01), consisting mainly of high-high value clusters (H–H) and low-low value clusters (L-L). In 2011, the H-H cluster was in Yunnan Province in southwestern China, the high values surrounded by low values cluster (H-L) appeared in Beijing, and the L-L cluster was in Heilongjiang Province in northeastern China. In 2018, the H-H cluster was still in Yunnan province, and the L-L clusters were distributed in Heilongjiang, Jilin, and Liaoning province, covering the whole northeastern region. From the perspective of spatial and temporal distribution, tourism demand formed a growth pole in southwestern China centered on Yunnan from 2011 to 2018, and tourism demand in Guizhou, adjacent to Yunnan, grew significantly and showed a spatial diffusion effect the region (Fig. 7). While in northeast China, the number of L-L clusters increased, and the provincial growth rate of tourism demand in low-value agglomeration was much lower than the national average during the study period. The existence of the H-L cluster in Beijing in 2011 and the disappearance of this cluster in 2018 indicated that Beijing had strong competitiveness in the region in the early stage, and the weakening polarization effect and the increasing diffusion effect diminished in the later stage when tourism demand was gradually distributed in a balanced manner in the Beijing-ring region. It suggested that tourism demand was more stable in China spatially in high-value clustering, and low-value clustering had increased, forming an increasingly stable high-value area in the southwest and low-value area in the northeast. Therefore, tourism demand in one province largely influences tourism demand in the adjacent provinces.

### Driving forces of tourism demand

#### Influencing factors of tourism demand

**Figure 8:**
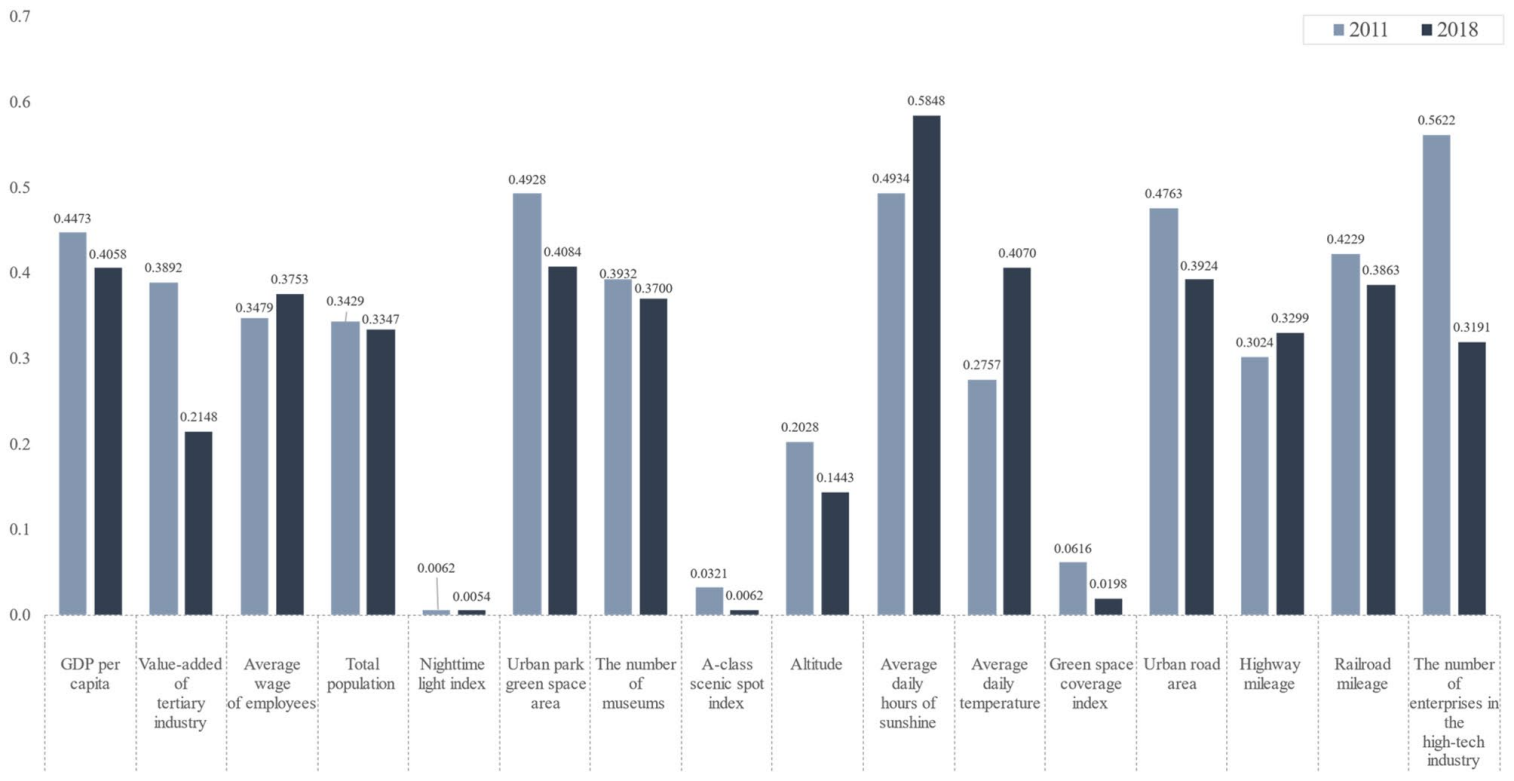
Power of determinant Q-statistic value for each driving factor in 2011 and 2018.

Figure 8 showed the explanatory power of the driving factors for tourism demand in 2011 vs. 2018. In 2011, The number of enterprises in the high-tech industry (0.5622) had the highest explanatory power, implying that GDP had a remarkably noticeable impact on tourism demand. Average daily hours of sunshine (0.4934), Urban park green space area (0.4928), Urban road area (0.4763), GDP per capita (0.4473), Railroad mileage (0.4229) had the same level of high explanatory power, which meant that these three drivers had the most noticeable impact on tourism demand. The number of museums (0.3932), value-added of tertiary industry (0.3892), Average wage of employees (0.3479), Total population (0.3429), Highway mileage (0.3024) were also significant drivers of tourism demand. Average daily temperature (0.2757) and altitude (0.2028) also influenced tourism demand. Green space coverage index (0.0616), A-class scenic spot index (0.0321). The nighttime light index (0.0062) had minimal explanatory power on tourism demand.

In 2018, the average daily hours of sunshine (0.5848) significantly affected tourism demand, expressing the strongest association with tourism demand: urban park green space area (0.4084), Average daily temperature (0.407). GDP per capita (0.4058) had a significant explanatory power on the spatial distribution of tourism demand. Urban road area (0.3924), Railroad mileage (0.3863), Average wage of employees (0.3753), The number of museums (0.37), Total population (0.3347), Highway mileage (0.3299), The number of enterprises in the high-tech industry (0.3191) were also significant factors influencing tourism demand. Tourism demand was limitedly influenced by the value-added of tertiary industry (0.2148), Altitude (0.1443). Green space coverage index (0.0198), A-class scenic spot index (0.0062). The nighttime light index (0.0054) had minimal effects on tourism demand.

#### Interaction of driving factors

One hundred twenty couple of interactions were generated yearly between the 16 factors in 2011 and 2018, the bulk of which had an enhancing effect on tourism demand, with the primary interaction type being nonlinear enhancement (55.83% in 2011 and 75.83% in 2018), followed by bi-variable enhancement (43.33% in 2011 and 23.33% in 2018). The explanatory power of the interaction on tourism demand was greater than that of the single factor with the maximum explanatory power.

**Figure 9:**
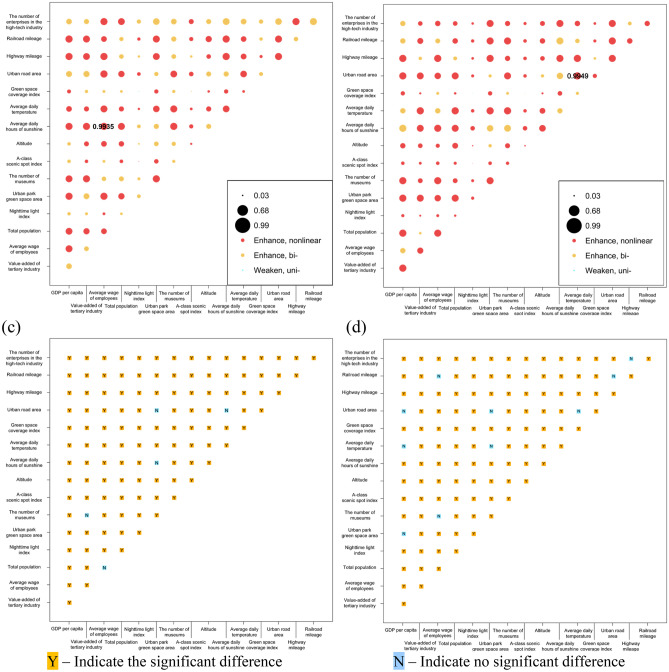
GeoDetector results: Power of determinants in interaction in (**a**) 2011 and (**b**) 2018; the difference of the impacts between two explanatory variables in (**c**) 2011 and (**d**) 2018.

As shown in Fig. 9a,c, in 2011, Average wage of employees-Average daily hours of sunshine (0.9935), Average wage of employees-Urban road area, GDP per capita-Railroad mileage, were two-factor non-linearly enhanced interactions with Q-statistic for the interactions greater than 0.99, showing a tourism demand with extreme explanatory power. Urban park green space area-Highway mileage, Average daily hours of sunshine-Highway mileage, Average daily hours of sunshine-Railroad mileage, Average wage of employees-Highway mileage, The number of museums-Average daily hours of sunshine, Average wage of employees-Urban park green space area, Urban park green space area-The number of museums were two-factor none-linearly enhanced interactions with interaction Q-statistic values greater than 0.98, a significant increase in the influence of synergy on tourism demand.

In 2018 (Fig. 9b,d), Average daily temperature-Urban road area (0.9949), Urban park green space area-Average daily temperature, Average daily temperature-Highway mileage, Total population-Average daily temperature, GDP per capita-Highway mileage, Average wage of employees-Highway mileage, GDP per capita-Total population, The number of museums-Average daily temperature, were two-factor non-linearly augmented interaction patterns with interaction Q-statistic greater than 0.99, which almost wholly control the spatial distribution of tourism demand. Value-added of tertiary industry-Average daily temperature, GDP per capita-Average daily hours of sunshine, GDP per capita-Value added of tertiary industry, GDP per capita-Urban road area, GDP per capita-The number of museums, GDP per capita-Urban park green space area, were two-factor none-linearly enhanced and GDP per capita-Average daily hours of sunshine was two-factor enhanced with interaction Q-statistic values greater than 0.98.

The regional economic development and construction were the main drivers, followed by the size of the population and the base of tourism services, and again by the traffic conditions, with the influence of natural factors and tourism resources being minimal in 2011. Moreover, by 2018, the influence of tourism comfort factors began to rise, such as average daily hours of sunshine and average daily temperature, representing a significant increase. The level of social and personal economic development and transportation conditions also increased influence to 2011. It indicated that in the aftermath of the world economic crisis and during the economic recovery, the driving force affecting tourism demand is the city's economy and level of development. However, after high economic growth, tourists have more requirements for the comfort of the experience during tourism, and the economy is no longer the main driving force directly affecting the spatial distribution of tourism demand.

#### Interaction mechanism

**Figure 10:**
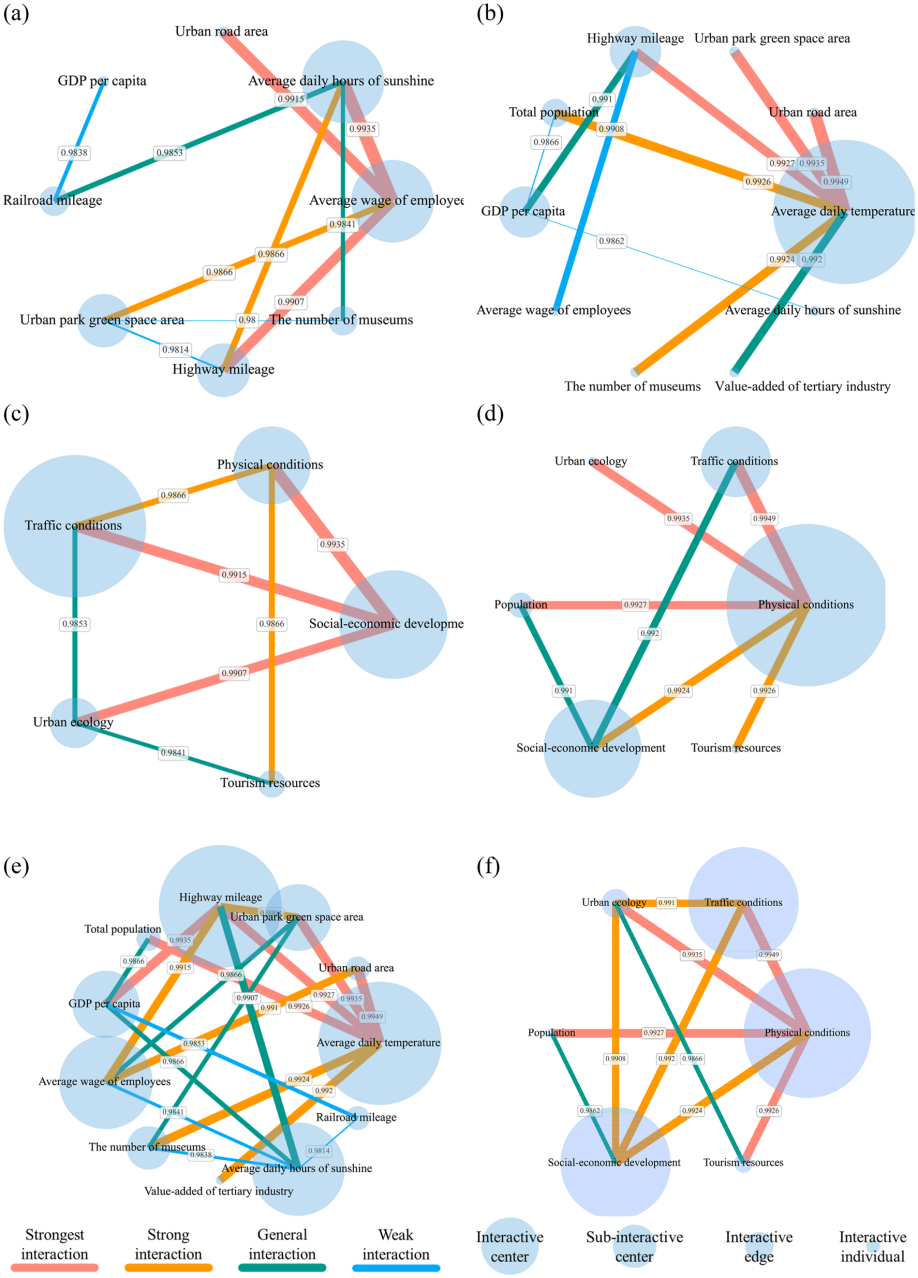
Diagram of Interactive Network: the interactive network of proxy variables in 2011 (**a**) and 2018 (**b**); interactive network of dominants in (**c**) 2011 and (**d**) 2018; (**e**) global interactive network based on proxy variables; (**f**) global interactive network based on dominants.

We filtered the five combinations with the maximum Q-statistic values from each of the 120 interaction combinations for 2011 and 2018, respectively, with an average explanatory power greater than 0.99 and two-factor nonlinear enhancement. It indicates that these combinations play a decisive role in the spatial distribution of tourism demand. The dominant factors to which each proxy variable belongs are also shown in Table 1. The ten combinations generated the interaction networks with the most explanatory power. The proxy variables and the determinants are mapped as nodes; the cumulative value of the Q statistics for the interactions between the node and other nodes determines the node's size. The interactions between the factors are edges, and the Q-statistic values for interaction measured the weight of the edges. Different hierarchical interaction networks are visualized in Fig. 10 revealed the interactive mechanism.

**Table 1:** Top 5 Q statistic values of interactive detection in 2011 and 2018.

Year	Rank	Interaction	Q for interaction
2011	1	The average wage of employees-average daily hours of sunshine (social-economic development-physical conditions)	0.9935
2011	2	The average wage of employees-urban road area (social-economic development-traffic conditions)	0.9915
2011	3	GDP per capita-railroad mileage (social-economic development-traffic conditions)	0.9907
2011	4	Urban park green space area-highway mileage (urban ecology-traffic conditions)	0.9866
2011	5	Average daily hours of sunshine-highway mileage (physical conditions-traffic conditions)	0.9866
2018	1	Average daily temperature-urban road area (physical conditions-traffic conditions)	0.9949
2018	2	Urban park green space area-average daily temperature (urban ecology-physical conditions)	0.9935
2018	3	Average daily temperature-highway mileage (physical conditions-traffic conditions)	0.9927
2018	4	Total population-average daily temperature (population-physical conditions)	0.9926
2018	5	GDP per capita-highway mileage (social-economic development-traffic conditions)	0.9924

From the perspective of the interaction of proxy variables, a strong triangular network community was formed in 2011 by average wages of employees—average daily hours of sunshine-highway mileage. In 2018, the interaction network shaped a significant polarization with the average daily temperature at the center, and average daily temperature and highway mileage formed a chain community. From the perspective of the interaction of dominants, Fig. 10c,d illustrate a substantial triangular network community formed by traffic conditions dominated by socio-economic development conditions and complemented by physical conditions in 2011. This community continued to persist in 2018, with the difference that the roles of physical and traffic conditions were switched.

Notably, the most significant interaction results in 2011 and 2018 were integrated to reveal the driving mechanisms impacting the distribution of tourism demand. Physical conditions existed at the core of the interaction network, mainly in the form of average daily temperature, which interacted extensively with other factors and was the central driver influencing the distribution of tourism demand, indicating that tourism comfort is the basis of the natural scenery tourism attraction. Socio-economic development followed closely behind in physical conditions, with the average wage of employees representing the general level of economic development in the region and the prosperity of the tertiary sector, characterizing the level of tourism services, as well as being the foundation for driving the city to be a tourist attraction. The importance of traffic conditions was uncovered in tourism accessibility and the compression of the time distance. The three formed a concrete network of interactions that influenced the spatial distribution of tourism demand.

## Discussion

Tourism is one of the critical engines of local economic development and serves as a regulatory tool for coordinated development within and between countries and regions. Tourism is extraordinarily vital and dynamic, and according to the latest report of the World Tourism Organization, tourism worldwide has shown a rapid recovery after the impact of COVID-19. This paper suggested a theoretical framework to explore the spatial distribution of driving tourism demand based on a spatially stratified heterogeneity perspective, obtained the dominant drivers to shape the spatial distribution of tourism demand, and discussed the interaction mechanisms among the drivers.

Internet Big data have derived a tremendous amount of Internet operation records of individual Internet users, which provide us with new means of observation. Early observations of tourism demand relied on management statistics of scenic spots and cities. After 2008 Internet search engines, big data were widely used as indicators to quantify tourism demand, and they proved to have good reliability at different geographic scales to accurately reflect the amount of tourism demand. For example, Yang et al. and Xin et al. predicted tourism demand in Hainan Province^36^, China, and Beijing Forbidden City^37^, Beijing, China. The results proved that the Baidu index could more accurately reflect tourism demand's spatial and temporal characteristics. This paper uncovered the spatial distribution of tourism demand and flow network patterns reflected by the Baidu index on a national scale (Figs. 3, 6). It demonstrated that the Baidu index could characterize tourism demand dynamically and build tourism flow networks.

Notably, there were regional differences in the distribution of inter-provincial tourism demand in China. The study results showed that tourism demand increased significantly from 2011 to 2018, the spatial clustering pattern of tourism demand was not randomly distributed (Fig. 5); there were two types of spatial effects in regional tourism growth, namely spatial spillover and spatial heterogeneity^21^. The high tourism demand cluster was shaped in the southwest, and the low tourism demand cluster was rendered in the northeast (Fig. 7). The spatial competitive effect of high and low imbalance in the capital ring gradually vanishes, and the tourism demand in the central region tends to be homogeneous.

**Figure 11:**
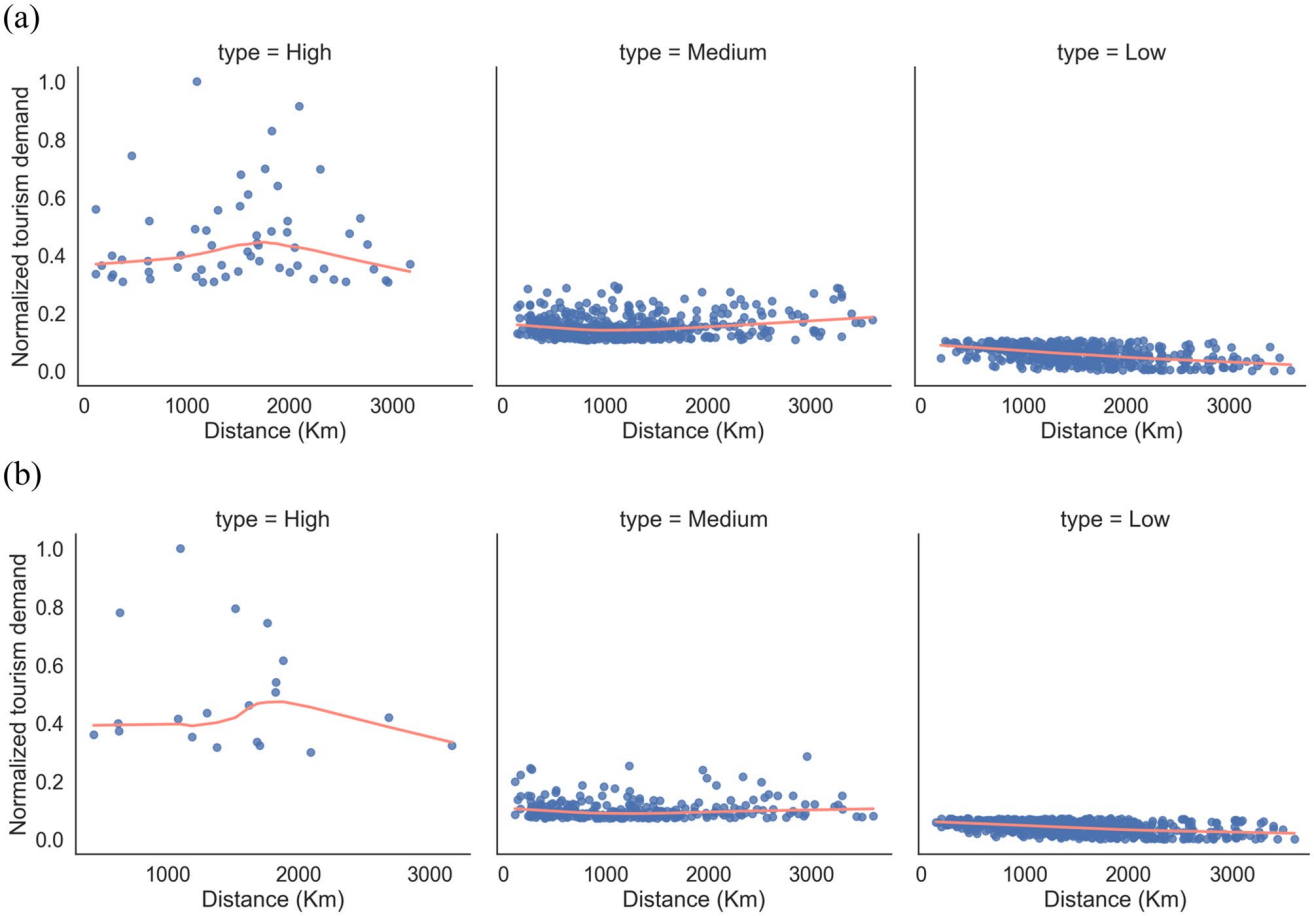
Results of locally weighted regressions of distance and travel demand: (**a**) 2011 (**b**) 2018. 2011 and 2018 tourism demand flow intensities were normalized separately. Locally weighted regression^38^ of distance and normalized tourism demand intensity was performed according to the stratification of tourism demand flows in Fig. 5, where the Euclidean distances of flows were calculated in the Beijing_1954_3_Degree_GK_Zone_35 coordinate system.

By investigating the spatial distribution pattern of domestic inter-provincial tourism demand in China, we recognized heterogeneity in the sensitivity of long-distance tourism flows to the distance in different intensities (Fig. 11). There was an explicit reversal at 1900 km for high-intensity tourism flows, i.e., the distance between origin and destination was shorter than 1900 km, and tourism demand positively corresponded with distance; conversely, whereas the was over 1900 km, tourism demand declined with increasing distance. Medium-intensity tourism flows were not sharp with distance. Low-intensity tourism flows obeyed the distance decay law.

The dominants that have a decisive influence on tourism demand were physical conditions, socio-economic development, and traffic conditions; the proxy variables are the average daily temperature, the average wage of employees, and highway mileage.

Physical conditions had high explanatory power for the spatial distribution of tourism demand, proving that natural scenery tourism was more prevalent in China than urban humanistic tourism. Tourist attraction and comfort of the natural scenery type were determined physical conditions; for instance, world natural heritage sites have a stronger role in promoting tourism^39^. Murphy et al. analyzed daily time-scale park visitation and weather data for Pinery Provincial Park, Canada, from 2000 to 2009, demonstrating the high sensitivity of tourism demand to average daily temperatures^40^.

While socio-economic development furnishes the foundation for breeding urban humanistic tourism, the average wage of employees is an efficient indicator of the region's economic development^41^, where high-quality tourist reception, available public information, travel safety, and diversified recreational convenience services are important factors attracting tourists. Meanwhile, efficient administrative supervision services in economically developed regions directly impact public information, recreational convenience, safety protection, and recreational convenience^42^.

Traffic conditions make it possible to connect tourists to tourist attractions. Wang et al. (2020) uncovered a well-coupled relationship between tourism efficiency and traffic accessibility in Hubei Province^43^, China, from 2011 to 2017. Highway mileage enhanced the coverage of inter-regional connections and compressed tourists' time costs to their destinations; on the other hand, it also increased the polarization of intra-regional connections, thus benefiting the central regions rather than the peripheral ones from the traffic^44^. This finding was consistent with the results that Southwest China forms a high tourism demand cluster, while Northeast China is a low tourism demand cluster in “Spatial dependency” section and Fig. 7. Another recent study^45^ has observed that less-developed central and western regions attract more visitors than developed eastern regions by improving transportation conditions in China.

Several aspects need to be considered in related follow-up studies. First, this study analyzed the drivers of tourism demand at the provincial level in China, with prominent medium- and long-distance tourism characteristics. In contrast, complete tourism demand occurs between prefecture-level cities, which should be considered the primary research unit in the future. However, writing a crawler program to request raw data from the Baidu index to obtain the daily tourism demand O-D flow has limitations. Therefore, moderately reducing the scope of the study may be helpful. Secondly, direct dominants in this study's theoretical framework of the driving mechanism are economic development level, population size, urban ecological conditions, tourism resources, natural environment, transportation conditions, and science and technology innovation. The results showed that the geographical variables represented by the tourism resources index, night lighting index, and green space coverage index have little impact on tourism demand, it might be caused by the difference between the scale of rasters and spatial panel data, these rasters may have more efficiently representation on the scale of urban. Therefore, more representative ones should be selected in future research as proxy variables. Finally, the COVID-19 global pandemic significant public safety event on tourism demand is also very impacting. The effects of severe public contingencies and the government's immediate response policies on tourism demand should be added to the tourism demand driving mechanism in the future.

## Conclusions

This study adopted Baidu index data spatialized into flow space, and multi-source data to investigate domestic tourism demand's spatial pattern and drivers during China's rapid economic development (2011–2018). The results show that (1) China's domestic tourism demand has significantly increased, shaping a spatial pattern in which first-tier cities and western regions where the core tourism destinations and the tourism attractiveness of northeastern regions gradually disappeared. The tourism demand network is increasingly prosperous and gradually develops from disorderly to orderly, with eastern regions as the main source of tourists. (2) From the single driving factor, the factor with the strongest and increasing control over the spatial distribution of tourism demand is sunshine hours > the average wage of employees > highway mileage. (3) In terms of the composite factor interaction results, the interaction network formed by physical conditions-economic development level-transportation conditions steadily and strongly determines the spatial distribution pattern of tourism demand.

The novelty of this study is the flow-based spatialization of the search engine index (Baidu index), which efficiently mapped the spatial mode of tourism demand and unearthed the network formed by domestic tourism flows, domestic long-distance travel in China is positively correlated with distance in terms of travel demand between the source and destination within 1900 km and vice versa. Additionally, the factors affecting the spatial distribution of tourism demand were interpreted from spatial heterogeneity, and the significant impact of the interaction between factors on tourism demand was resolved and captured the complex network. The findings of this paper can provide a reference for regional tourism planning decision-makers. Simultaneously, it can also provide a systematic tourism demand driving mechanism for tourism demand forecasting researchers and promote modeling accuracy.
